# The Cumming's Patent

**Published:** 1872-02

**Authors:** 


### EDITORIAL DEPARTMENT.

---

*The Cumming's Patent.*—The Dentists throughout the country are forming organizations to contest the validity of the Cumming's patent on Vulcanite Base, and an appeal from the decision of the U. S. District Court of New York, (reaffirming the validity of this patent previously sustained in the Wetherbee case) brought by Benoni E. Gardiner in the Supreme Court of the U. S. will be heard during the present month (February,) and the matter decided.

So confident are the leading members of the profession throughout the country that the Cumming's patent cannot be sustained after June next, at which time the Goodyear patent expires, that they are determined to use every effort to obtain an impartial hearing.

To do this successfully will require united action, and they therefore invite all practitioners of dentistry to co-operate.

The proprietors of the Cumming's patent, represented by Josiah Bacon, will spare no amount of money and energy in the attempt to sustain their claim, and it is therefore necessary for every dentist who regards their claims as unjust, to contribute as much as he is able in assisting those who are endeavoring to remove this great burden from the profession.

The following from Dr. S. S. White in the *Cosmos*, will enlighten those who are uninformed on this subject:

"Desiring to give a reply that will not mislead those asking for information and advice, I applied to one who, by reason of his knowledge of the patents and of the proceedings under them, is quite competent to give a statement of the existing condition of the case, and an opinion as to the course of action open to dentists in the present dilemma. The following is his statement:

"The Nelson Goodyear patent for hard rubber will expire in May, 1872. It can be extended for a further term by special act of Congress, but there is no probability that such legislation can be procured for the continuance of a patent owned by purchasing speculators—and which has paid so largely. It is safe to conclude that that will be the end of it.

The Cumming's patent, which claims the present mode of making hard rubber artificial dentures, was granted June 7, 1864, for the term of seventeen years.

The specification and claims of the amended and reissued Cumming's patent are such that, while it stands as a valid patent, a dentist who makes hard rubber or vulcanite work cannot evade it. He must either conceal that he does it, or procure a license from its owners, or accept the issue and fight them in the U. S. Courts.

Heretofore the suits brought against dentists infringing this patent have resulted in its favor. Many persons attribute these results to anything but the soundness and goodness of the patent. We hear of partiality of the judges, collusive consent of the defendants, etc. Yet whatever may be the truth of these rumors, the fact is becoming apparent that by several judgments in its favor, this Cumming's patent will acquire such a standing as will give its owners great facility in the courts for taking injunctions against infringers.

It is the announced rule of the Boston Dental Vulcanite Company to license dentists under both the Goodyear and Cumming's patents, and in no other way.

They do not grant licenses for less than one year.

If therefore any dentist makes vulcanite during the year 1872, he must conceal it perfectly, or expect to pay Josiah Bacon's Company, the price of a license etc., for the year.

If he vulcanizes a single case he will be liable, if it be previous to May 6th, on both patents, and after that date on the Cumming's patent only.

To resist the Goodyear patent at this late day would be folly

To resist the Cumming's patent is wise if it be well done. Whoever does it must calculate to do it alone, and at his own expense, or know for himself the aid which others will give him.

There is a good deal of talk and inquiry at this time, but there does not exist any organized association for general defense or to aid individual resistants.

It is possible to break the Cumming's patent. It is an un ound and defective patent. To break it is quite as possible for an individual as for an association, but to do it will require the knowledge, the pluck, and the money to carry it through.

The individual dentist who knows he has not got these of himself or is not sure to get them from others, had better take a license—when compelled to do so—or let vulcanite alone.

On the whole, this last is the very best way. Pass the word around, *let Vulcanite alone.* Do not make a bit of it this year ; be ready to show and prove that you have not ; use any other material known to the profession as being suitable ; be firm in this course for a short time.

Cut off the resources of the Bacon party, give them no hitch for a claim or a suit against you ; carry out this abstinence until June next, when they will be able to attack with the Cumming's patent only. Organize in the meantime a strong association, retain the counsel which defended the Wetherbee case ; add to them Hon. Samuel Fisher, late patent commmissioner, and any other eminent counsel demanded by local interests to inspire full confidence in the effort and protect individuals from malicious prosecution ; make up a full, perfect case, and break up this swindle."

